# Chronic Elevation of Liver Enzymes in Acute Intermittent Porphyria Initially Misdiagnosed as Autoimmune Hepatitis

**DOI:** 10.4061/2011/392049

**Published:** 2011-01-18

**Authors:** A. González Estrada, S. García-Morillo, L. Gómez Morales, P. Stiefel García-Junco

**Affiliations:** Internal Medicine, Hospital Universitario Virgen del Rocío, 41013 Seville, Spain

## Abstract

Autoimmune hepatitis is a disease characterized by an elevation of liver enzymes, as well as specific autoantibodies. It is more common in women than men. We describe a 32-year-old woman with elevated transaminases, autoantibodies, and a liver biopsy result suggestive of autoimmune hepatitis. The indicated treatment was administered without showing a satisfactory response. The patient had a family history of acute intermittent porphyria (AIP) so we decided to begin treatment with hematin, achieving a complete remission of the symptoms. Acute intermittent porphyria is a rare condition characterized by neurovisceral symptoms, abdominal pain being the most common of them. The disease has a higher prevalence among young women and certain European countries such as Sweden, Great Britain, and Spain. A correct diagnosis and prompt treatment are essential because patients affected by AIP must have a strict followup due to the fatal outcome of the outbreaks.

## 1. Introduction

Acute intermittent porphyria (AIP, also known as Swedish porphyria) is a rare autosomal dominant inherited metabolic disorder. The underlying cause is a deficiency in the porphobilinogen deaminase (PBG) enzyme, which is the third enzyme in the heme biosynthesis pathway. The prevalence of a mutant AIP gene may be as high as 1 per 500, thus it shows an incomplete penetrance; the prevalence of symptomatic disease is only 1-2 per 100,000, as stated by Badminton and Elder [1]. This disease is more frequent among young women. Although most individuals never develop symptoms, the neurovisceral crises are the most common presentation pattern in this type of porphyria, abdominal pain being the most characteristic symptom. Autoimmune hepatitis (AIH) is a chronic liver disease of unknown etiology, characterized as periportal hepatitis with hypergammaglobulinaemia and positive autoantibodies. It can be prompted by environmental or inherent factors. Antibodies to nuclei (ANA), smooth muscle (SMA), and soluble liver antigen/liver pancreas (SLA/LP) characterize type 1 AIH. As discussed elsewhere, type 2 AIH is associated with antibodies to liver/kidney microsomes (ALKM-1) and antibodies to liver cytosol antigen (ACL-1 or LC1) [2, 3]. The indicated treatment for AIH is steroids with or without Azathioprine; this is typically well tolerable although some patients need to switch the treatment to Cyclosporine or Mycophenolate mofetil as published by Muratori et al. [4]. We describe a case of AIP, previously misdiagnosed as AIH, in a patient with persistent elevation of liver enzymes.

A 32-year-old woman was referred to our hospital with a diagnosis of AIH, with no clinically relevant background or influence of any medication or alcohol use. She had persistent elevation of transaminases (three times normal values), gammaglobulines 18.8%, IgA 571 mg/dL, IgG 1440 mg/dL, albumin 51.5%, and albumin/globulin ratio 0,95%, as well as an elevation of positive smooth muscle antibodies (1/160 and 1/80), and we excluded all other possible causes of hepatitis, including viral markers (HBsAg and Anti-HCV), alpha 1 antitrypsin 136 mg/dL, plasma iron 47 *μ*g/dL, and copper 157 *μ*g/dL. A liver biopsy was performed which denoted a chronic hepatitis with moderate periportal activity, showing lymphocyte inflitrates in the portal space that exceeds the parenchimal stoma interphase. The hepatocytes were arranged in a trabecular manner, and there was no evidence of histologic alterations except for minor swelling. There were also some fibrous porto-portal tracts. The revised Original Scoring System of the International Autoimmune Hepatitis Group was applied, reaching a score of 15 points, which makes it a probable diagnosis of autoimmune hepatitis as reported by Manns et al. [5] 

We started her on Prednisone 1 mg/kg, and Azathioprine 50 mg per day, increasing it to 150 mg per day; however, there was no response to the treatment, and the liver enzymes remained elevated by three times the normal value (see Table 1). The only significant aspect of the medical history was that three out of her five sisters had been diagnosed with AIP. Following this information, we tested for AIP, and the laboratory results showed urine coproporohyrin 433 *μ*g/day (normal range 50–200 *μ*g/day) and uroporphyrin 173 *μ*g/day (normal range 5–70 *μ*g/day). Erythrocyte deaminase PBG enzymatic activity was 42 U/LH (normal range 85–160 U/LH). Urine porphobilinogen (12 mg/day) and *δ*-aminolaevulinic acid (7.4 mg/day) were slightly increased, as well as a sharply defined plasma fluorescence emission of 619 nm. Therefore, the diagnosis of AIP was established. 

We began treatment with 250 mg daily for four days of hematin (NORMOSANG), following a monthly dose, along with a gradual reduction of the steroids until suspension. New laboratory tests were performed two months later which included hemogram, basic and liver profiles, cholesterol, triglycerides, TSH, coagulation, antinuclear, antimitochondrial, anti-ALKM, anti-SMA, and celiac disease antibodies, proteinogram, and immunoglobulins. All test results showed values within normal limits, except for the following: ALT 40 UI/l, seroalbumins 56.1%, alpha 2 globulins 10.3%, beta globulins 12.4%, and total proteins 6.9 g/dL.

The available data makes the diagnosis of autoimmune hepatitis unlikely, mostly because of the response obtained when the new treatment was established. The patient was reevaluated for the following years and remained asymptomatic, without presenting abdominal pain or dark urine, and having normal menstrual periods and laboratory test values.

## 2. Discussion

The coexistence of AIP and AIH is extremely rare; they do, however, share a chronic elevation of transaminases. This association has not yet been described. We have conducted a thorough research through the literature looking for such association but were unable to find a link. Because of this, we conclude that the resolution of this case report has shown that the final diagnosis was acute intermittent porphyria, expressed as an elevation of transaminases, and was initially misdiagnosed as AIH, a situation that could have led the patient to an increase in both morbidity and mortality. As reported by Ohtani et al., there have been reports of acute intermittent porphyria causing a mild increase of the liver enzymes and others that report an elevation of transaminases associated with abdominal pain [6, 7]. With this knowledge, it is imperative to denote that an early diagnosis of AIP is crucial because acute porphyries can be life threatening, and, of all of them, AIP is considered to be the most severe. As presented by Raigal, a 50% reduction of the activity of the PBG deaminase is sufficient to appreciate clinical manifestations [8]. As suggested elsewhere, it is also important to keep in mind all the known triggering factors, such as medication drugs, changes in women's hormonal cycles, caloric deprivation, tobacco, alcohol, or infections [9]. These factors can cause the accumulation of delta aminolevulinic acid (ALA) and PBG—which are normally eliminated through urine and feces—in the tissues, producing the clinical symptoms. AIPs treatment is based on avoidance of the attacks. During an acute attack, intravenous carbohydrates (400 g per day) should be administered, as well as hematin 3–5 mg per kg for at least 4 days as stated by Raigal et al. [8]. In order to have a better understanding of the possible association between both diseases, more studies have to be performed. 

In conclusion, young women with chronic elevation of transaminases of unexplained etiology and unspecific liver biopsy should be evaluated for AIP especially if they also present intense and recurrent abdominal pain and dysautonomy, as the possibility of such a diagnosis is latent. Also, periodic screening of alpha-fetoprotein should be performed in patients with AIP and chronic elevation of liver enzymes since they have greater risk of developing hepatocarcinoma.

## Figures and Tables

**Table 1:** 

	Initial situation	After steroids and azatioprine	After hematin
AST/ALT	81 U/L/96 U/L	85 U/L/86 U/L	34 U/L/40 U/L
GGT/FA	12 U/L/177 U/L	10 U/L/165 U/L	10 U/L/122 U/L
Gammaglobulines	18.8%	18.7%	17.7%
Ig A	571 mg/dL	497 mg/dL	331 mg/dL
SMA	1/160	1/80	Negative

AST: aspartate aminotransferase, ALT: alanine aminotranferease, GGT: gamma glutamyl transferase, FA: alkaline phosphatase, and SMA: smooth muscle antibodies.
